# Abundance and sinking of particulate black carbon in the western Arctic and Subarctic Oceans

**DOI:** 10.1038/srep29959

**Published:** 2016-07-15

**Authors:** Ziming Fang, Weifeng Yang, Min Chen, Minfang Zheng, Wangjiang Hu

**Affiliations:** 1 State Key Laboratory of Marine Environmental Science and College of Ocean and Earth Sciences, Xiamen University, Xiamen 361102, China

## Abstract

The abundance and sinking of particulate black carbon (PBC) were examined for the first time in the western Arctic and Subarctic Oceans. In the central Arctic Ocean, high PBC concentrations with a mean of 0.021 ± 0.016 μmol L^−1^ were observed in the marginal ice zone (MIZ). A number of parameters, including temperature, salinity and ^234^Th/^238^U ratios, indicated that both the rapid release of atmospherically deposited PBC on sea ice and a slow sinking rate were responsible for the comparable PBC concentrations between the MIZ and mid-latitudinal Pacific Ocean (ML). On the Chukchi and Bering Shelves (CBS), PBC concentrations were also comparable to those obtained in the ML. Further, significant deficits of ^234^Th revealed the rapid sinking of PBC on the CBS. These results implied additional source terms for PBC in addition to atmospheric deposition and fluvial discharge on the western Arctic shelves. Based on ^234^Th/^238^U disequilibria, the net sinking rate of PBC out of the surface water was −0.8 ± 2.5 μmol m^−3^ d^−1^ (mean ± s.d.) in the MIZ. In contrast, on the shelves, the average sinking rate of PBC was 6.1 ± 4.6 μmol m^−3^ d^−1^. Thus, the western Arctic Shelf was probably an effective location for burying PBC.

Black carbon (BC), including charcoal, soot and graphitic carbon^1^, is the product of incomplete combustion of biomass and fossil fuels^2^. Recently, BC has been raising concern owing to its influence on carbon cycling^3^,^4^. The annual formation of BC from biomass burning is estimated to be 50–270 Tg, and 6.4–28 Tg is emitted into the atmosphere^5^,^6^, accounting for an important fraction of the refractory carbon in the environment^2^. Although about 80% of BC derived from biomass burning remains in the soil^6^, recent studies suggest that an amount of BC leaves the soil via leaching or erosion^7^,^8^,^9^. Dissolved BC (DBC) makes up around 10% of the dissolved organic carbon (DOC) in river waters^10^. Soot-BC, the most refractory BC, accounts for up to 50% of the bulk particulate organic carbon (total-POC) pool in seawater^11^, and up to 35% of organic carbon in sediments^12^. Globally, 26 Tg of particulate BC (PBC) and 26.5 Tg of DBC are discharged into the oceans each year^10^,^13^. This riverine BC, especially DBC, becomes active when exposed to the sunlit surface water, and some of it can decompose into carbon dioxide under solar irradiation^14^,^15^. Soot-BC is also reported to decrease DOC concentrations and promote the aggregation of organic materials^16^. In addition, PBC can lead to an overestimate of biogenic POC in seawater, since traditional measurements of POC include the PBC fraction although it is not biogenic^11^,^17^. PBC has a different δ^13^C signature from marine biogenic POC, and so incorporating PBC in the POC origin-assessing model leads to different results than those from an earlier two-endmember model (i.e. terrestrial and marine biogenic endmembers)^17^. Thus, BC is important to an understanding of global carbon cycling.

Although the input of BC into the oceans has been preliminary quantified, the removal or loss of BC from seawater is only rarely investigated. Stubbins *et al*.^14^, based on controlled experiments, are the first to report the photo-degradation of DBC in natural seawater; and Flores-Cervantes *et al*.^11^ the first to estimate the sinking of soot-BC out of the water column using the ^234^Th/^238^U technique. Both studies indicate that the removal processes of BC from seawater are of great importance for our understanding of its role in carbon cycling, such as DOC turnover and POC sedimentation. Therefore, extensive research is needed to examine the removal of BC from seawater. Theoretically, approaches used for estimating the sinking of particles, e.g. ^234^Th/^238^U, ^210^Po/^210^Pb, and the sediment trap^18^,^19^,^20^,^21^, would be applicable to PBC sinking quantification. Since PBC is rarely determined in seawater^11^,^17^,^22^, these methods have been little used to quantify the sinking of PBC, except for a report on the Gulf of Maine^11^.

The Arctic Ocean is potentially an important field for studying BC on a global scale. Although the atmospheric deposition of BC might be low owing to the low intensive anthropogenic activities surrounding the Arctic Ocean^23^, few studies indicate the interesting cycling of BC in the Arctic Ocean. A preliminary estimate indicates that > 200 Gg C of soot-BC are discharged into the coastal seawater from Pan-Arctic rivers annually^13^, accounting for about 5% of the fluvial POC. This fluvial BC could continuously increase as a result of the increasing coastal erosion induced by global warming^24^,^25^. In addition, the increasing melting of permanent sea ice during summer also releases more atmospherically-deposited BC into the Arctic Ocean^26^. These observations suggested that BC cycling in the Arctic may have much closer relations with global warming than in mid-latitudes. Thus, it is necessary to investigate the geochemical behavior of BC in the Arctic Ocean. Here, we present the abundance of PBC determined using the chemo-thermal oxidation (CTO) method, i.e. CTO-375^27^, from 31°N in the North Pacific Ocean to 82°N in the Arctic Ocean. The CTO-375 method preferentially quantifies the highly condensed soot-type portion of BC and is appropriate for soot determination in marine sediments^28^. We used the ^234^Th/^238^U technique to quantify the sinking rates of PBC. Comparisons between the Arctic central basin (and Subarctic shelves) and the mid-latitudes were examined to illustrate the distinctive behavior of PBC in the western Arctic and Subarctic Oceans.

## Results and Discussion

### PBC abundance in the marginal ice zone (MIZ), the Chukchi and Bering Shelves (CBS) and the mid-latitudinal Pacific Ocean (ML)

**Figure 1:**
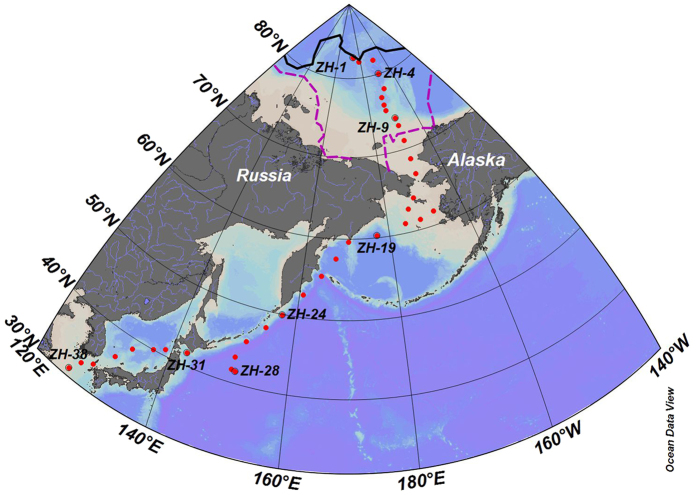
Sampling stations in the western Arctic and Subarctic Ocean. This map was generated in Ocean Data View (v. 4.7.4, https://odv.awi.de/). Solid line and dashed line denote the ice edge on Sep. 1 and Aug. 1 respectively (data from NOAA, http://polar.ncep.noaa.gov/seaice/Historical.html).

**Figure 2:**
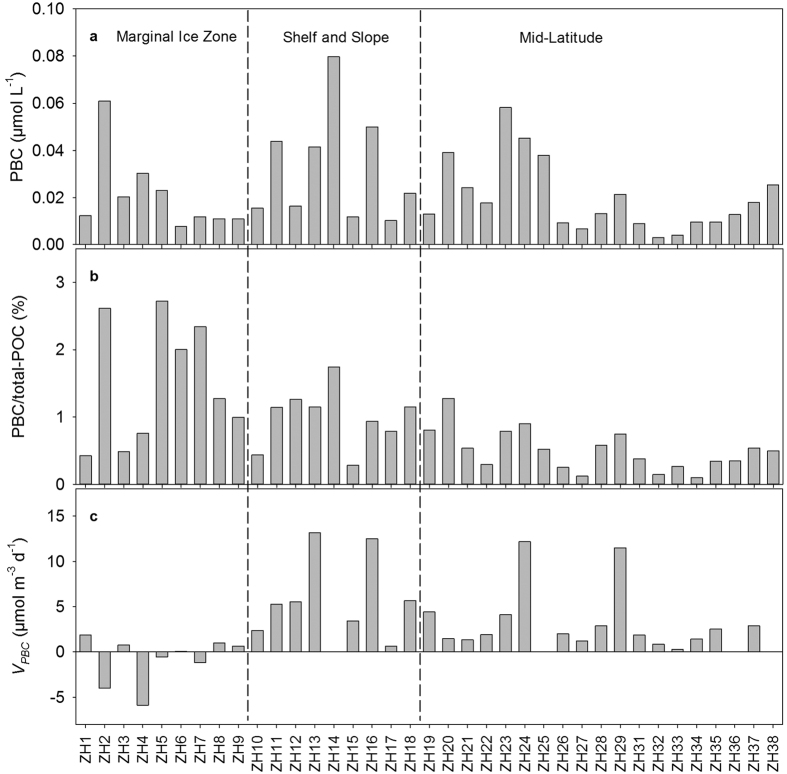
Latitudinal patterns of (**a**) PBC concentration, (**b**) ratio of PBC to the total-POC, and (**c**) input/sinking rate of PBC (*V*_*PBC*_).

In the central Arctic Ocean (Fig. 1), the concentrations of PBC ranged from 0.008 to 0.061 μmol L^−1^ at the MIZ stations, averaging 0.021 ± 0.016 μmol L^−1^ (Table 1). PBC concentrations varied from 0.010 μmol L^−1^ to 0.080 μmol L^−1^ with an average of 0.032 ± 0.023 μmol L^−1^ on the CBS (Fig. 2). Statistically, the concentrations of PBC from the MIZ did not show a difference from the PBC concentrations of < 0.003–0.058 μmol L^−1^ obtained at the ML stations (*t*-test with *p* > 0.05, Table 2). Atmospheric deposition is reported to be the main source term of PBC to the open ocean^17^. East Asia is the largest emission region of BC^29^. The few studies available report that the content of BC in the atmosphere over the Japan-Bering Sea ranges from 14–160 ng m^−3^, and is less than 14 ng m^−3^ from the Bering Sea to 85°N in the western Arctic Ocean^30^, indicating that atmospheric deposition of BC is much higher in the ML than the Arctic Ocean. Thus, other processes, in addition to atmospheric deposition, are responsible for the comparable concentrations between the ML and MIZ.

**Figure 3:**
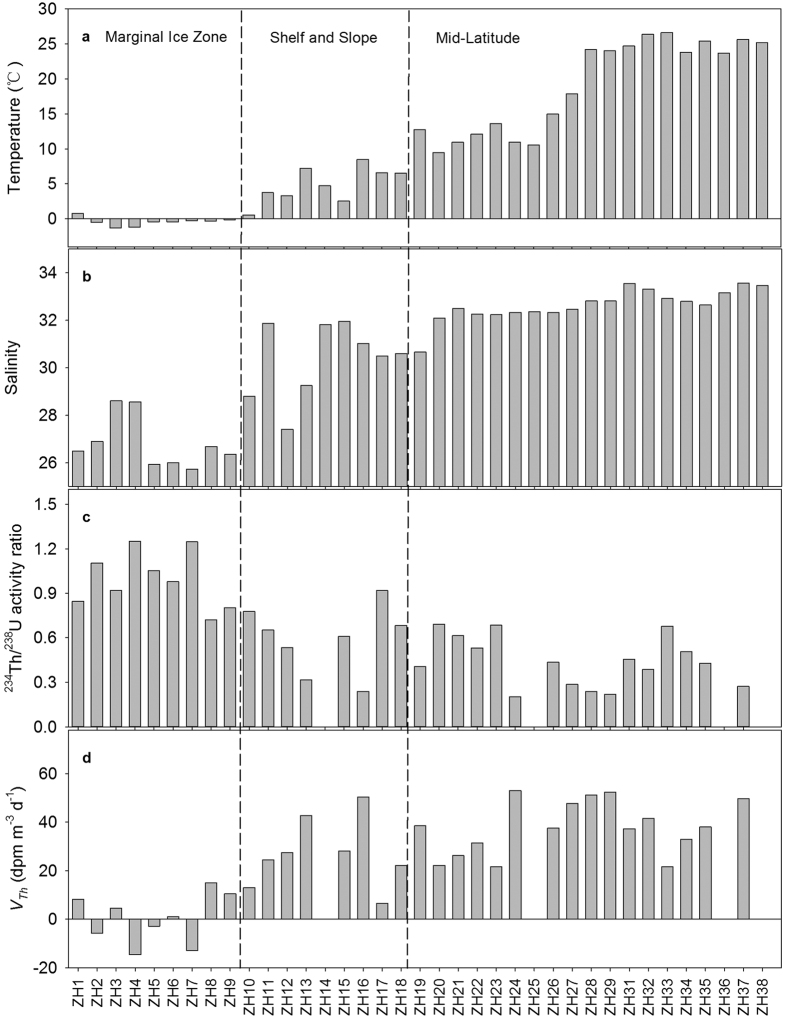
Latitudinal patterns of (**a**) temperature, (**b**) salinity, (**c**) disequilibria between ^234^Th and ^238^U, and (**d**) input/sinking rate of ^234^Th (*V*_*Th*_) to show the significantly different characteristics of the marginal ice zone.

Three processes were proposed to be responsible for the high PBC levels in the central Arctic Ocean (Fig. 2a). First, faster sinking of PBC in the ML than the Arctic Ocean might be the crucial process resulting in the comparability between the MIZ and ML. Based on the ^234^Th/^238^U ratios, the particle sinking rate in the ML (avg.: 38.9 ± 11.8 dpm m^−3^ d^−1^) was much higher than in the Arctic Ocean (avg.: 0.4 ± 10.3 dpm m^−3^ d^−1^) (*t*-test, *p* < 0.001, Fig. 3). Secondly, the release of accumulated atmospherically deposited PBC on the sea ice during the cold months would increase the concentration of PBC at the MIZ stations. This process was supported by atmospheric deposition of ^7^Be in the Arctic Ocean, which accumulates on the sea ice during October-July^31^. Based on the datasets developed by the National Aeronautics and Space Administration team using satellite passive microwave radiances^32^, the sea ice concentrations were more than 40% at ZH1 and ZH2, and sea ice at other stations in the MIZ almost melted from 20 August to 3 September. With the sea ice melting, accumulated soot-BC would be released into the surface waters. In our study, temperatures of surface water at the MIZ stations were around freezing point, varying from −1.31 to 0.80 °C with an average of −0.42 ± 0.61 °C (Fig. 3a). Salinity varied from 25.74 to 28.61, averaging 26.81 ± 1.07 (Fig. 3b). Low temperature and salinity showed significant difference from either the CBS or the ML stations (Table 2), illustrating the evident influence of sea ice melting at the MIZ stations. Lastly, the across-shelf particle flow resulting from the sediment-laden sea ice would also contribute to the increasing concentration of PBC at the MIZ stations. The formation of sea ice along the coast and on the western Arctic shelves with shallow water depths is widely found to entrain a large amount of sediment^33^. The transport of sediment-laden sea ice to a large degree dominates the sediment regime in the Arctic Ocean^34^ and pollutant dispersal^35^. In our study, sea ice transported sediment was illustrated by the ^234^Th/^238^U ratios. At the MIZ stations, most of the ^234^Th/^238^U ratios were more than unity (Fig. 3c). Usually, this ratio is less than unity in open surface seawater because ^234^Th is particle reactive and quickly removed with particles comparing with its grandparent of ^238^U, resulting in a deficit of ^234^Th to ^238^U. The excess of ^234^Th suggested an exogenous source of ^234^Th in addition to ^238^U decay generation. Similar excess ^234^Th is also reported in the same region during summer 2003, which is used to reflect the ice-rafted sediment^36^. Since the shelf sediments contain relatively high PBC contents^13^,^37^, the sediment-laden ice could transfer entrained PBC to the central Arctic Ocean, contributing to the high PBC concentrations during the ice-melting time window.

**Figure 4:**
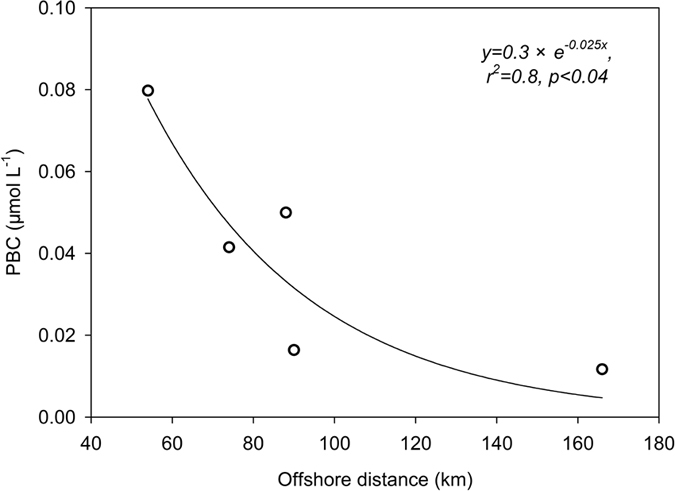
Descending PBC concentrations offshore on the Chukchi and Bering shelves.

The CBS also showed higher sinking rates of particulate matter relative to the MIZ (Fig. 3d). However, its soot-BC concentrations were comparable to the MIZ. Sea ice did not seem to explain this scenario since PBC released from the ice sunk to deep water after ice melting, which would result in low PBC. There must be other PBC source terms beside sea ice melting. Spatially, PBC exponentially decreased offshore with an attenuation constant of 0.025 ± 0.009 km^−1^ within 200 km (Fig. 4), similar to observations in all the coastal environments examined^11^,^17^,^38^. This spatial pattern lent support to the terrigenous trait of PBC^39^ in the western Arctic shelves. Based on the locations (Fig. 1), most of the CBS stations were influenced by river water from the Yukon, Mackenzie, and/or the East Siberian Rivers. Using the S-δ^18^O^40^ and S-δ^18^O-PO_4_^*^ tracers^41^, river water accounted for 5–10% of the bulk seawater for the shelf stations. The low salinity (averaging 30.36 ± 1.57) for the shelf stations (Table 2) also illustrated the river water influence. Based on the evaluation of a fluvial discharge of PBC from the Pan-Arctic Rivers^13^, the Mackenzie River, representing the main contributor, accounted for almost half of the total arctic fluvial PBC, followed by the East Siberian Rivers. The Mackenzie River water is transported to the Chukchi Shelf stations studied via the Beaufort Sea current^40^. Although the Yukon River discharge of PBC is less than that of the Mackenzie River^13^, its river water signals are clearly identified at most stations north or south of the Bering Strait^40^. Combining these studies, the Pan-Arctic and Yukon Rivers could discharge 228 ± 55 Gg PBC per year to the western Arctic shelf regions.

### Ratios of PBC to total-POC at the MIZ, CBS and ML stations

At the MIZ stations, PBC accounted for 0.43–2.72% of the total-POC (Fig. 2b) with a mean of 1.51 ± 0.92% (Table 2), which was higher than the observed average of 0.50 ± 0.30% at the ML stations (*t*-test, *p* < 0.05). Such a difference was attributed to the lower POC concentrations at the MIZ stations (averaging 1.90 ± 1.49 μmol L^−1^) than those of the ML (averaging 4.19 ± 2.19 μmol L^−1^). However, the ratio of PBC to total-POC was lower than those reported in coastal and lake waters, such as 0.34–4.83% (averaging 2.45%) in the northern Gulf of Mexico^17^, 1–20% in the Gulf of Maine^11^, 3.9–8.9% in Lake Superior^42^, and 1.9–17% for the Mississippi River^43^. In the traditional evaluation of biogenic POC sinking out of surface waters, PBC is not excluded^44^, because of the lack of PBC data, although PBC is not biogenic POC. Hence, the POC sinking rates would be overestimated. Our study revealed that PBC accounted for only < 3% of the total-POC, indicating that PBC did not evidently impact previous POC sinking results in the central Arctic Ocean. However, it should be noted that the influence of PBC on the sinking of POC in the intermediate and deep water would become more and more important owing to its increasing contribution downwards^17^. Since POC collected in deep water is usually used to evaluate the transport efficiency of phytoplankton fixed carbon dioxide from the surface ocean to deep water, such an increase of PBC in POC pool would lead to more overestimate of this efficiency with the increase of depth.

### Sinking rates of PBC in the MIZ, CBS and ML

During sea ice melting, the sinking rates of PBC varied from −5.87 to 1.85 μmol m^−3^ d^−1^ (Table 3) with a mean of −0.8 ± 2.5 μmol m^−3^ d^−1^ (Table 2) at the MIZ stations, implying an overall input of PBC. Similarly, weak sinking of particle-reactive Th is observed in the Arctic Basin^45^. In contrast, the ML showed much higher PBC sinking rates, averaging 3.3 ± 3.5 μmol m^−3^ d^−1^ (*t*-test, *p* < 0.01, Table 2), although its PBC concentrations were comparable to the MIZ stations. The very low sinking of PBC during sea ice melting was ascribed to the freshwater as identified by the very low salinity and temperature (Fig. 3). Owing to the low salinity of the surface water, the upper water column was significantly stratified, which inhibited the sinking of particles. Thus, PBC, mainly from low and middle latitudes via atmospheric transport^46^,^47^,^48^, remained suspended for a long time in the upper ocean during sea ice melting.

For the CBS stations, the ^234^Th/^238^U ratios varied from 0.28–0.89 with a mean of 0.59 ± 0.23 (Table 2). Compared with the central Arctic stations, the CBS showed significant deficits of ^234^Th (*t*-test, *p* < 0.05), indicating rapid sinking of ^234^Th and particulate matter. Thus, much higher sinking rates of PBC could be expected. Indeed, the sinking rates of PBC ranged from 0.65 to 13.16 μmol m^−3^ d^−1^ (Fig. 2), averaging 6.1 ± 4.5 μmol m^−3^ d^−1^ (Table 2). On the one hand, all shelf stations exhibited net sinking of PBC, quite different from the MIZ stations. On the other hand, the sinking rates were higher than the value observed in the MIZ (*t*-test, *p* < 0.05), implying that the western Arctic shelf was a very effective region for burying PBC. This view was supported by the contrasting PBC contents in the suspended particulate matter (SPM) and sediments. The ratios of PBC to SPM in weight (Table 1) varied from 0.06 to 0.16% with an average of 0.11 ± 0.05% on the Chukchi Shelf, and from 0.02 to 0.10% with an average of 0.06 ± 0.03% on the Bering Shelf. In sediments, the reported average contents of PBC are 0.18 ± 0.09% for the Chukchi and 0.09 ± 0.07% for the Bering Shelf 37. The effective reservation of PBC in the sediments in the western Arctic and Subarctic Shelf is ascribed to its refractory nature^17^,^39^,^49^. For instance, the ratios of PBC to total-POC varied from 0.28 to 1.74%, averaging 0.99 ± 0.44% at the shelf stations (Table 2), while these ratios increase to 8.7–73.7% with a mean of 19.3 ± 15.3% in the CBS sediments^37^. Taken together, these results indicated that the shelf sediments would serve as crucial archives of PBC in the western Arctic Ocean.

### Preliminary budget of PBC on the western Arctic Shelf

**Figure 5:**
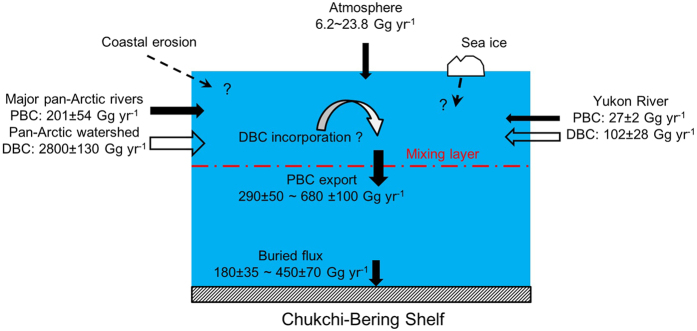
Budget of PBC on the Chukchi and Bering shelves.

In spite of limited stations, we managed to estimate the crude fluxes of PBC out of the surface ocean over the western Arctic shelves. This estimate was a necessary first step in budgeting PBC in the western Arctic Ocean. For simplicity, the box model was adopted. All published PBC data were collected for the calculation (Table 4). Atmospheric deposition to the Arctic region is about 60–230 Gg yr^−1^ 48,50,51. Combining the area of the CBS (1.6 × 10^6^ km^2^), atmospheric input was estimated to be 6.2–23.8 Gg yr^−1^ for the CBS (Fig. 5). The PBC discharge from the Yukon, Mackenzie, and East Siberian Rivers (Lena, Indigirka and Kolyma) is 228 ± 55 Gg yr^−1 ^13,52. Based on the above studies and given the CBS seawater, it seemed that there are multiple sources of PBC. In our study, PBC sinking fluxes were estimated either for the ice-free duration or for all year round, in order to preliminarily set a broad range. Usually, ice-free time lasts for about 4 months in the Chukchi Sea and 6 months in the Bering Sea^53^. Since ^234^Th was only analyzed in surface waters, the sinking of both ^234^Th and PBC is estimated out of the upper 15 m, which is the minimum mixed depth for the shelf stations (Table 3). Similar depth is also adopted by *Carrizo and Gustafsson*^54^ during their evaluation of the inventory of Polychlorinated Biphenyls in the upper mixed layer on the western Arctic Shelf. Thus, the fluxes represent their low limits. The estimated sinking flux was 290 ± 50 Gg yr^−1^ for the CBS during the ice-free period (Fig. 5). If the atmospheric deposition is the predominant input of PBC, this estimate would largely reflect the real sinking of PBC, since atmospheric deposited soot-BC in ice-covered months also sinks within the ice-free period. However, if other sources contribute a large amount of PBC all year round, sinking of PBC would also occur within the ice-covered period. Thus, the sinking flux of PBC estimated would be 680 ± 100 Gg yr^−1^. This value represented an overestimate due to the fact that atmospherically deposited PBC does not sink in ice-covered months. The broad range of PBC sinking fluxes indicated that extensive work is required to understand the PBC budget in the western Arctic Ocean.

Since more than a half of the riverine PBC is removed within 200 km off the river mouth^17^, the total contribution of atmospheric deposition and riverine discharge seemed to be much less than the low limit of PBC sinking. It appeared that other sources were important to account for the PBC sinking. One of the probable sources was the ice-rafted sediments, which were entrained during sea ice formation in shallow water and then transported to the shelves with currents and/or winds after the ice broke. The excess of ^234^Th to ^238^U as observed during ice melting in the MIZ (Fig. 3) lent support to this hypothesis. The second potential source of PBC was expected to relate to the coastal erosion, which is enhanced via ongoing global warming^24^,^25^. Approximately 44 Tg of old carbon is discharged into the East Siberian Arctic Shelf each year^55^. The final possible source may be the incorporation of riverine DBC into the SPM^3^,^22^. The fluvial DBC from the major Pan-Arctic rivers (i.e. the Ob’, Yenisey, Lena, Kolyma, Mackenzie and Yukon) and the whole Pan-Arctic watershed are estimated to be 1500 ± 130 and 2800 ± 300 Gg yr^−1^, respectively^56^, which may have significant influence on the budget of PBC on the western Arctic shelves. However, these indicative sources of PBC have not yet been well constrained. More studies are needed to examine the influence of coastal erosion and the ice-raft on the input of PBC into the Arctic Ocean.

Flores-Cervantes *et al*.^11^ report that two-thirds of the PBC exported out of seawater eventually reaches the sediments. Assuming the same efficiency, the buried PBC could be 180–450 Gg yr^−1^ for the CBS (Fig. 5). These results were lower than that of 200–800 Gg yr^−1^ in the Gulf of Maine^11^,^57^, and 1100 Gg yr^−1^ on the northern European Shelf 58. However, they were comparable to the Brazilian and Argentinian continental shelves^12^. Therefore, the western Arctic Shelf could not be neglected in quantifying global PBC cycling.

## Conclusions

PBC in seawater was examined for the first time in the western Arctic and Subarctic Oceans, and also compared with mid-latitude regions. Atmospheric deposition and fluvial discharge were the main sources of PBC in the surface waters of the Arctic Ocean. Sinking rate, to a large extent, controlled the abundance of PBC. During sea ice melting periods, the release of atmospheric deposited PBC on the sea ice also had an important influence on the abundance of PBC in the surface waters. In summer, PBC concentrations between the MIZ, CBS and mid-latitudes appeared to be comparable to each other. A preliminary estimate indicated additional PBC sources beside atmospheric deposition and fluvial discharge, such as ice-rafted material, coastal erosion, and the adsorption of DBC onto particles. Both ^234^Th deficits and PBC sinking rates implied a very effective sedimentation of PBC on the western Arctic shelf and the subarctic shelves.

## Material and Methods

### Sample collection

Surface water samples were collected at 37 stations during the 5th Chinese Arctic Research Expedition (CHINARE) cruise on board R/V *XUELONG* from 3 to 23 September, 2012 (Fig. 1). This cruise provided an opportunity for examining the variability in BC over a large spatial scale from mid-latitude in the northern hemisphere (31 °N) to high latitude in the Arctic Ocean (82 °N). During sampling, the sea ice was melting. Thus, some stations were positioned along the ice melting margins, which enabled us to note the influence of sea ice melting on PBC. Based on salinity, temperature and geography, the stations were divided into three groups in order to reveal the regional patterns of PBC, i.e. the MIZ (Stations ZH-1 to ZH-9), the CBS (Stations ZH-10 to ZH-18), and the ML (Stations ZH-19 to ZH-38). The ML stations, representing the region greatly influenced by anthropogenic activities, were chosen to compare with Arctic and subarctic stations in order to illustrate the influence of PBC on the North Polar regions with less anthropogenic activities.

At each station, more than 40 L of seawater were collected at around 1 m using a Ruttner water sampler. Part of the seawater was immediately filtered through a pre-combusted (450 °C for 4 h) QMA filter membrane with a pore size of 1.0 μm (Whatman^TM^) in order to collect particulate matter for determination of PBC and particulate ^234^Th. To collect as many particles as possible, 15–40 L of seawater was required at each station, depending on their SPM contents. The collected particulate samples were desalinated with Milli-Q water, and dried at 60 °C. The dissolved ^234^Th in four liters of filtrate were concentrated using the MnO_2_ co-precipitation technique^45^. Briefly, NH_4_OH was added to adjust the pH value of the filtrate to 9.0. Then, KMnO_4_ and MnCl_2_ solutions were sequentially added while stirring. After settling for 6 h, the MnO_2_ precipitate was collected using a QMA filter. The precipitation conditions (e.g. filter membranes, pH) were examined in detail using calibrated Th spikes to obtain a stable yield of ^234^Th in our laboratory. The filters, carrying ^234^Th and MnO_2_, were dried at 60 °C.

### ^234^Th and SPM measurements

All samples, carrying particulate and dissolved ^234^Th, were counted using a low background *β* counter. After 5 months, all samples were recounted for evaluating the counts of other non-^234^Th/^234^ ^m^Pa beta emitters^45^. The specific activities of ^234^Th in the present study were corrected to the sampling time. The errors presented here were propagated from the statistical counting errors of ^234^Th. ^238^U was calculated based on salinity data from calibrated CTD and the widely used relation between ^238^U and salinity^59^. The concentrations of SPM were determined based on the difference in weight between the filter membrane and the total weight including SPM and filter.

### PBC and POC measurements

After ^234^Th determination, the particulate samples were used to determine POC and PBC. The filters were fumigated with concentrated HCl for 48 h to remove carbonate and dried at 60 °C prior to analysis. POC content was measured using one eighth of the filter membrane and a CHN analyzer (Elementar, Germany). The sampling protocols of the aliquot are widely used for determining multiple parameters on a piece of membrane^45^,^60^. In brief, the aliquot of membrane used for POC determination was subsampled using a stainless steel cutter with a diameter of 15 mm, thus the cutting area was 12.2% of the total area used for collecting particles. BC samples were pre-treated using the CTO-375 method^17^,^27^. The protocol used removes the less labile fractions in the BC continuum and leaves mainly soot, which is appropriate for quantifying soot-BC in marine sediment^28^. To date, only a few studies on PBC in seawater are reported; and the CTO-375 method is usually adopted^11^,^17^. Using the same method enabled our results to be compared with published data.

In brief, the de-carbonated filter was combusted at 375 °C in the presence of air for 24 h before CHN analysis. Residual carbon in the filters after combustion using the CTO method was mainly defined as soot-BC, which is the most refractory portion of the BC spectrum^1^,^28^. The National Institute of Standards and Technology standard 1941b was used to check the analytical processes for both POC and PBC. The resulting POC and PBC values in our analysis were 3.01 ± 0.07% (n = 4) and 0.52 ± 0.03% (n = 5), which were similar to the results in a previous study^17^.

### PBC sinking rate estimate using ^234^Th/^238^U disequilibria

In summer, sea ice melting showed rapid temporal variability in the Arctic Ocean. Given the widely used methods for constraining the sinking of particles (i.e. ^234^Th/^238^U, ^210^Po/^210^Pb, and sediment trap)^18^,^19^,^20^,^21^, the ^234^Th/^238^U method was the favorite option because its half-life matched the timescale of ice melting. In seawater, ^234^Th is produced by ^238^U disintegration. Owing to its particle-reactive nature, ^234^Th is readily adsorbed onto particulate matter, and quickly sinks into deep water with the particles. As a consequence, ^234^Th in the surface ocean is usually at a deficit relative to ^238^U. The extent of ^234^Th deficiency represents its sinking rate with the particles. Based on the similar sinking manner between ^234^Th and PBC, the sinking rates of PBC can be estimated using ^234^Th. To date, the only other study on PBC sinking, which is reported for the Gulf of Maine, proves the validity of the ^234^Th/^238^U disequilibrium technique for quantifying PBC sinking^11^.

We used ^234^Th/^238^U to evaluate the sinking rate of PBC in a manner similar to those for quantifying the cycling of PBC^11^ and POC^18^,^44^. In brief, the sinking rate (*V*_*PBC*_) was calculated from the equation:


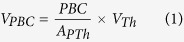


where *V*_*PBC*_ and *V*_*Th*_ represent the sinking rate of PBC and ^234^Th from the surface ocean in μmol C m^−3^ d^−1^ and dpm m^−3^ d^−1^, respectively; *A*_*PTh*_ is the activity concentration of particulate ^234^Th in dpm L^−1^; and PBC is the concentration of PBC in μmol L^−1^. The sinking rate of ^234^Th (i.e. *V*_*Th*_) was estimated from the widely used box model in a steady-state^18^:


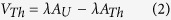


where *λ* is the decay constant of ^234^Th (0.02876 d^−1^); and *A*_*U*_ and *A*_*Th*_ represent the activity concentrations of ^238^U and the total ^234^Th in dpm L^−1^, respectively. Since an acoustic Doppler current profiler was not used in the present study, the advection term could not be presented separately. Therefore, it was combined with the export term, and expressed as *V*_*Th*_. Thus, *V*_*Th*_ included all input and export terms in the model box. The positive values of *V*_*Th*_ corresponded to the net sinking of ^234^Th, and negative *V*_*Th*_ represented the net input of ^234^Th (Table 3).

## Additional Information

**How to cite this article**: Fang, Z. *et al*. Abundance and sinking of particulate black carbon in the western Arctic and Subarctic Oceans. *Sci. Rep.* **6**, 29959; doi: 10.1038/srep29959 (2016).

## Figures and Tables

**Table 1:** Activity concentrations of dissolved ^234^Th (^234^Th_D_), particulate ^234^Th (^234^Th_P_), total ^234^Th (^234^Th_T_), ratios of the total ^234^Th to ^238^U and the concentrations of PBC, POC, and SPM in surface water.

Station	Longitude (°E)	Latitude (°N)	POC	PBC	^234^Th_P_	^234^Th_D_	^234^Th_T_	Th/U	SPM
			(μmol L^−1^)		(dpm L^−1^)				(mg L^−1^)
ZH-1	173.94	82.71	2.90	0.012	0.05 ± 0.02	1.54 ± 0.33	1.59 ± 0.33	0.85	0.25
ZH-2	178.75	82.07	2.33	0.061	0.09 ± 0.02	2.02 ± 0.33	2.11 ± 0.33	1.10	0.22
ZH-3	−169.01	81.92	4.20	0.020	0.12 ± 0.02	1.74 ± 0.33	1.87 ± 0.33	0.92	0.21
ZH-4	−168.99	80.00	3.98	0.030	0.08 ± 0.02	2.46 ± 0.33	2.53 ± 0.33	1.25	0.14
ZH-5	−169.00	78.00	0.84	0.023	0.12 ± 0.02	1.81 ± 0.33	1.94 ± 0.33	1.05	0.03
ZH-6	−171.99	77.00	0.39	0.008	0.16 ± 0.02	1.64 ± 0.34	1.80 ± 0.34	0.98	0.02
ZH-7	−171.99	76.00	0.51	0.012	0.13 ± 0.02	2.14 ± 0.33	2.27 ± 0.33	1.25	0.11
ZH-8	−172.00	75.25	0.86	0.011	0.17 ± 0.02	1.20 ± 0.33	1.37 ± 0.33	0.72	0.15
ZH-9	−169.02	74.00	1.10	0.011	0.18 ± 0.02	1.32 ± 0.34	1.50 ± 0.34	0.80	0.13
ZH-10	−168.97	73.00	3.58	0.016	0.09 ± 0.02	1.50 ± 0.33	1.59 ± 0.33	0.78	0.12
ZH-11	−168.86	71.00	3.82	0.044	0.20 ± 0.04	1.20 ± 0.30	1.41 ± 0.30	0.65	0.38
ZH-12	−168.86	68.62	1.30	0.016	0.08 ± 0.03	0.91 ± 0.29	0.99 ± 0.29	0.53	0.36
ZH-13	−168.91	66.72	3.61	0.041	0.13 ± 0.04	0.45 ± 0.29	0.58 ± 0.30	0.32	0.71
ZH-14	−171.83	63.96	4.58	0.080	n.d.	n.d.	n.d.	n.d.	0.99
ZH-15	−173.92	62.80	4.15	0.012	0.10 ± 0.05	1.19 ± 0.30	1.28 ± 0.30	0.61	0.86
ZH-16	−167.72	61.69	5.35	0.050	0.20 ± 0.05	0.24 ± 0.29	0.44 ± 0.29	0.24	1.15
ZH-17	−171.58	61.20	1.29	0.010	0.10 ± 0.03	1.83 ± 0.30	1.93 ± 0.30	0.92	0.23
ZH-18	−175.53	61.13	1.90	0.022	0.09 ± 0.04	1.31 ± 0.29	1.40 ± 0.30	0.68	0.46
ZH-19	176.86	60.26	1.61	0.013	0.11 ± 0.03	0.72 ± 0.29	0.83 ± 0.29	0.41	0.38
ZH-20	169.92	59.63	3.06	0.039	0.59 ± 0.04	0.91 ± 0.30	1.50 ± 0.30	0.69	0.53
ZH-21	167.00	57.56	4.50	0.024	0.47 ± 0.04	0.92 ± 0.26	1.39 ± 0.27	0.61	0.31
ZH-22	164.06	55.32	6.01	0.018	0.29 ± 0.04	0.90 ± 0.26	1.19 ± 0.26	0.53	0.28
ZH-23	160.73	52.78	7.38	0.058	0.30 ± 0.05	1.23 ± 0.26	1.53 ± 0.26	0.68	0.57
ZH-24	157.21	49.92	5.02	0.045	0.20 ± 0.04	0.25 ± 0.26	0.44 ± 0.26	0.20	0.99
ZH-25	154.68	47.94	7.28	0.038	n.d.	n.d.	n.d.	n.d.	0.99
ZH-26	151.79	45.64	3.71	0.009	0.18 ± 0.03	0.80 ± 0.26	0.98 ± 0.26	0.44	0.47
ZH-27	150.50	43.33	5.50	0.007	0.26 ± 0.03	0.38 ± 0.26	0.64 ± 0.27	0.29	0.48
ZH-28	151.10	41.56	2.27	0.013	0.24 ± 0.01	0.31 ± 0.26	0.54 ± 0.27	0.24	0.32
ZH-29	150.42	41.70	2.85	0.021	0.10 ± 0.02	0.40 ± 0.25	0.50 ± 0.25	0.22	0.43
ZH-31	142.53	41.61	2.33	0.009	0.18 ± 0.02	0.90 ± 0.30	1.08 ± 0.30	0.45	0.22
ZH-32	138.91	40.78	2.04	≤0.003	0.15 ± 0.02	0.76 ± 0.29	0.91 ± 0.29	0.39	0.17
ZH-33	137.03	40.04	1.51	≤0.004	0.29 ± 0.02	1.29 ± 0.30	1.58 ± 0.30	0.68	0.10
ZH-34	133.81	38.63	9.53	0.010	0.22 ± 0.04	0.96 ± 0.29	1.18 ± 0.30	0.51	0.72
ZH-35	131.76	36.46	2.82	0.010	0.15 ± 0.03	0.84 ± 0.29	0.99 ± 0.30	0.43	0.26
ZH-36	129.06	33.76	3.67	0.013	n.d.	n.d.	n.d.	n.d.	0.23
ZH-37	127.26	32.88	3.35	0.018	0.31 ± 0.04	0.34 ± 0.30	0.65 ± 0.30	0.27	0.16
ZH-38	125.84	31.12	5.11	0.025	n.d.	n.d.	n.d.	n.d.	0.58

**Table 2:** Comparisons of various parameters for the three sub-regions, including temperature, salinity, sea ice concentrations, PBC concentrations, and sinking/input rates of thorium and PBC.

Region	MIZ	CBS	ML	MIZ/ML^a^	CBS/ML^a^
T (°C)	−0.42 ± 0.61	4.86 ± 2.56	19.11 ± 6.63	−0.02 (<0.001)	0.25 (<0.001)
Salinity	26.81 ± 1.07	30.36 ± 1.57	32.64 ± 0.67	0.8 (<0.001)	0.9 (0.002)
Ice concentration (%)	0–48	0	0		
PBC (μmol L^−1^)	0.021 ± 0.016	0.032 ± 0.023	0.020 ± 0.015	1.1 (0.88)	1.6 (0.15)
PBC/total-POC (%)	1.51 ± 0.92	0.99 ± 0.44	0.50 ± 0.30	3.0 (0.011)	2.0 (0.011)
^234^Th/^238^U)_A.R._	0.99 ± 0.19	0.59 ± 0.23	0.44 ± 0.17	2.2 (<0.001)	1.3 (0.10)
*V*_*Th*_ (dpm m^−3^ d^−1^)	0.4 ± 10.3	26.9 ± 14.4	38.9 ± 11.8	0.01 (<0.001)	0.69 (0.077)
*V*_*PBC*_ (μmol m^−3^ d^−1^)	−0.8 ± 2.5	6.1 ± 4.5	3.3 ± 3.5	−0.2 (0.001)	1.8 (0.097)

^a^The values in parentheses are the *p* results from *t*-tests for the MIZ vs. ML stations and the CBS vs. ML stations assuming *α* = 0.05. Sea ice concentrations are calculated based on the datasets from http://nsidc.org/data.

**Table 3:** The sinking/input rates of ^234^Th, POC and PBC estimated from the ^234^Th/^238^U disequilibria.

Station	Mixed layer depth	*V*_*Th*_	*V*_*POC*_	*V*_*PBC*_
	m	dpm m^−3^ d^−1^	μmol m^−3^ d^−1^	μmol m^−3^ d^−1^
ZH-1	—	8.21 ± 9.55	434 ± 523	1.85 ± 2.22
ZH-2	—	−5.78 ± 9.61	−152 ± 255	−3.98 ± 6.66
ZH-3	—	4.59 ± 9.49	158 ± 327	0.76 ± 1.58
ZH-4	—	−14.60 ± 9.46	−774 ± 536	−5.87 ± 4.07
ZH-5	—	−2.88 ± 9.60	−20 ± 66	−0.54 ± 1.80
ZH-6	—	1.11 ± 9.69	3 ± 23	0.05 ± 0.46
ZH-7	—	−12.94 ± 9.43	−49 ± 36	−1.15 ± 0.85
ZH-8	—	15.07 ± 9.63	78 ± 51	0.99 ± 0.65
ZH-9	—	10.53 ± 9.71	63 ± 59	0.63 ± 0.58
ZH-10	17	13.01 ± 9.58	541 ± 423	2.36 ± 1.85
ZH-11	20	24.40 ± 8.64	458 ± 183	5.24 ± 2.09
ZH-12	—	27.39 ± 8.24	439 ± 197	5.53 ± 2.48
ZH-13	15	42.77 ± 8.56	1145 ± 411	13.16 ± 4.73
ZH-15	20	28.18 ± 8.64	1214 ± 785	3.42 ± 2.21
ZH-16	25	50.42 ± 8.40	1338 ± 386	12.49 ± 3.60
ZH-17	20	6.58 ± 8.62	82 ± 110	0.65 ± 0.87
ZH-18	24	22.08 ± 8.55	491 ± 320	5.65 ± 3.68
ZH-19	—	38.57 ± 8.39	550 ± 186	4.42 ± 1.50
ZH-20	—	22.16 ± 8.70	115 ± 46	1.46 ± 0.58
ZH-21	—	26.25 ± 7.65	250 ± 77	1.34 ± 0.41
ZH-22	—	31.39 ± 7.59	652 ± 179	1.92 ± 0.53
ZH-23	—	21.56 ± 7.59	523 ± 203	4.13 ± 1.60
ZH-24	—	53.08 ± 7.47	1353 ± 320	12.18 ± 2.88
ZH-26	—	37.62 ± 7.62	793 ± 200	1.99 ± 0.50
ZH-27	—	47.76 ± 7.67	1007 ± 198	1.23 ± 0.24
ZH-28	—	51.20 ± 7.63	493 ± 79	2.87 ± 0.46
ZH-29	—	52.44 ± 7.27	1538 ± 342	11.50 ± 2.56
ZH-31	—	37.32 ± 8.55	489 ± 124	1.86 ± 0.47
ZH-32	—	41.61 ± 8.47	580 ± 133	0.53 ± 0.12
ZH-33	—	21.60 ± 8.67	113 ± 46	0.07 ± 0.03
ZH-34	—	32.90 ± 8.50	1407 ± 454	1.42 ± 0.46
ZH-35	—	38.02 ± 8.52	735 ± 233	2.51 ± 0.80
ZH-37	—	49.71 ± 8.58	536 ± 115	2.87 ± 0.62

**Table 4:** Discharge of DBC and PBC into the Arctic Ocean from the Pan-Arctic Rivers and atmospheric deposition.

Source term	DBC	PBC	BC
	(Gg yr^−1^)	(Gg yr^−1^)	(Gg yr^−1^)
Major Pan-Arctic Rivers	1400 ± 130^56^,*	206 ± 54^13^,**	
Yukon River	102 ± 28^56^	27 ± 2^13^,^52^	
Whole Pan-Arctic watershed	2800 ± 300^56^		
Atmospheric deposition to CBS			6.2–23.8^48^,^50^,^51^

^*^DBC flux from the major Pan-Arctic rivers including the Ob’, Yenisey, Lena, Kolyma and Mackenzie.

^**^PBC flux from the major Pan-Arctic rivers including the Ob’, Yenisey, Lena, Kolyma, Mackenzie and Indigirka.
